# Monocyte-driven inflamm-aging reduces intestinal barrier function in females

**DOI:** 10.1186/s12979-024-00469-6

**Published:** 2024-09-30

**Authors:** Candice Quin, Jessica A. Breznik, Allison E. Kennedy, Erica N. DeJong, Catherine M. Andary, Sofya Ermolina, Donald J. Davidson, Jinhui Ma, Michael G. Surette, Dawn M. E. Bowdish

**Affiliations:** 1 Institute of Medical Sciences, School of Medicine, Medical Sciences and Nutrition, University of Aberdeen, Aberdeen, Scotland; 2 Department of Medicine, Faculty of Health Sciences, McMaster University, Hamilton, ON Canada; 3 McMaster Institute for Research on Aging, Hamilton, ON Canada; 4 McMaster Immunology Research Centre, Hamilton, ON Canada; 5 Firestone Institute for Respiratory Health, St. Joseph’s Healthcare Hamilton, Hamilton, ON Canada; 6 Institute for Regeneration and Repair, Centre for Inflammatory Research, University of Edinburgh, Edinburgh, Scotland; 7 Department of Health Research Methods, Evidence and Impact, McMaster University, Hamilton, ON Canada

**Keywords:** Aging, Inflammation, Inflamm-aging, Immune remodeling, Intestinal barrier dysfunction

## Abstract

**Background:**

The intestinal barrier encompasses physical and immunological components that act to compartmentalize luminal contents, such as bacteria and endotoxins, from the host. It has been proposed that an age-related decline of intestinal barrier function may allow for the passage of luminal contents into the bloodstream, triggering a low-grade systemic inflammation termed inflamm-aging. Although there is mounting evidence to support this hypothesis in model species, it is unclear if this phenomenon occurs in humans. In addition, despite being well-established that biological sex impacts aging physiology, its influence on intestinal barrier function and inflamm-aging has not been explored.

**Results:**

In this study, we observed sex differences in markers of intestinal barrier integrity, where females had increased epithelial permeability throughout life as compared to males. With age, females had an age-associated increase in circulating bacterial products and metabolites such as LPS and kynurenine, suggesting reduced barrier function. Females also had age-associated increases in established markers of inflamm-aging, including peripheral blood monocytes as well as TNF and CRP. To determine if impaired barrier function was driving inflamm-aging, we performed a mediation analysis. The results show that the loss of intestinal barrier integrity was not the mediator of inflamm-aging in humans. Instead, persistent, low-grade inflammation with age preceded the increase in circulating bacterial products, which we confirmed using animal models. We found, as in humans, that sex modified age-associated increases in circulating monocytes in mice, and that inflammation mediates the loss of intestinal barrier function.

**Conclusion:**

Taken together, our results suggest that higher basal intestinal permeability in combination with age-associated inflammation, increases circulating LPS in females. Thus, targeting barrier permeability in females may slow the progression of inflamm-aging, but is unlikely to prevent it.

**Supplementary Information:**

The online version contains supplementary material available at 10.1186/s12979-024-00469-6.

## Background

The persistent low-grade inflammation that increases with age (‘inflamm-aging’) is associated with numerous health conditions including diabetes mellitus, cancer, dementia and depression [1]. Inflamm-aging is characterized by an increase in inflammatory mediators such as interleukin (IL)-6, tumor necrosis factor -α (TNF), and C-reactive protein (CRP) in the serum and tissue [reviewed in Refs [2, 3]]. These mediators activate inflammatory signalling pathways, changing the local and systemic milieu into a non-resolving pro-inflammatory state, leading to DNA damage and tissue death over time. Individuals with higher than age-average levels of inflammatory mediators are more likely to face premature mortality [4], whereas lower than age-average inflammation predicts good health [5]. Understanding the causes of chronic, age-associated inflammation is therefore a prerequisite to developing novel therapeutic interventions to improve health and quality of life in older adults.

Defects in intestinal barrier function have long been associated with increased inflammation [6]. The intestinal barrier encompasses physical (i.e., the epithelium and mucus layer), biochemical (i.e., antimicrobial peptides), and immunological (i.e., macrophages and other immune cells) components, which act to compartmentalize luminal microorganisms from the host. One of the prevailing theories on the origins of inflamm-aging is that impaired intestinal barrier function results in the translocation of bacterial products and triggers inflammatory responses from innate immune cells [7, 8]. Studies in animal models have shown that barrier integrity is lost with age [9] and can contribute to a persistent rise in bacterial lipopolysaccharide (LPS) or its proxy, LPS-binding protein, in the blood of aged mice [10] and nonhuman primates [11]. Low doses of LPS are known to polarize monocytes towards pro-inflammatory phenotypes [12], which are believed to contribute to the inflammatory conditions that arise in mid- to late-life [13, 14]. Although this hypothesis is compelling, the alternative is equally likely wherein age-associated inflammation precedes and causes intestinal barrier dysfunction that ultimately results in LPS translocation. Cytokines such as TNF, IFNγ, and interleukins regulate tight junction integrity [15], and stimulation with TNF has been shown to increase gut permeability [16]. We have shown that aged mice deficient in TNF do not demonstrate increased intestinal permeability with age [10], suggesting that inflammation is a driver of impaired barrier integrity. Based on these findings, we postulate that age-related increases in inflammation precede intestinal barrier dysfunction.

Monocytes are a likely contributor to age-associated inflammation as they are the principal producers of proinflammatory cytokines that are characteristic of inflamm-aging, including TNF and IL-6 [17, 18]. Data from our laboratory and others indicate that monocyte subsets change with age in both mice [19, 20] and humans [21]. For instance, circulating Ly6C^high^ inflammatory monocytes increase with age in mice and express more of the chemokine receptor, CCR2 [20]. These monocytes, which are equivalent toCD14^+^CD16^−^ classical and CD14^+^CD16^++^ inflammatory monocytes in humans [22], produce higher levels of pro-inflammatory cytokines than their Ly6C^low^/non-classical counterparts, and as a result, are often associated with immunopathology [20, 23]. Beyond their potential role in inflamm-aging, age-associated changes in monocyte subsets may contribute to impaired intestinal barrier function. Circulating monocytes continuously replenish Tim-4^−^CD4^−^and Tim-4^−^CD4^+^ gut macrophages, a process that is critically dependent on the expression of CCR2 [24]. It is therefore a possibility that age-associated changes in CCR2-mediated monocyte recruitment of intestinal macrophages may disrupt barrier homeostasis; however, this has not yet been investigated.

Another striking gap in our understanding of the relationship between intestinal barrier function and inflamm-aging is the role of biological sex. An increasing number of clinical observations have revealed widespread differences in aging and age-related diseases by biological sex [25]. For instance, the life expectancy of females is 15% longer compared to males [26]; however, despite longer lifespans, females have higher rates of disability, dementia and frailty, resulting in prolonged suffering at end-of-life [27]. As a consequence, females collectively spend about 20% more years living with disability [26]. These marked differences in aging trajectories make it important to account for biological sex in aging research and discourages the consideration of biological sex as a confounder, which can lead to results that are not biologically relevant to either sex [28]. Establishing whether sex differences in intestinal barrier function and/or monocyte-remodeling exist with age will be crucial to tailor sex-specific therapeutic strategies.

Herein, we assessed the effects of sex and age on peripheral blood monocytes, inflammatory mediators, and non-invasive markers of intestinal barrier function in healthy, non-frail, human participants. We then explored whether age- and sex-associated changes in immunity could modulate intestinal barrier dysfunction or vice versa. We also considered the interactions of sex and age on these parameters in mice. Our data highlight the importance of biological sex as a determinant of intestinal barrier integrity, wherein females have increased intestinal permeability, independent of age. Our findings also suggest that in humans, increased intestinal permeability is not the mediator of inflamm-aging. Instead, impaired barrier function resulting in LPS translocation is likely a consequence of persistent, low-grade systemic inflammation with increasing biological age.

## Methods

### Recruitment of research participants

Research participants were recruited from the Greater Hamilton Area (Ontario, Canada) between November 2017 and January 2020. All protocols were approved by the Hamilton Research Ethics Board (#1949). The inclusion criteria encompassed individuals aged over 18 years who were willing and able to provide consent, biological samples, and a health questionnaire. Venous blood was drawn in anti-coagulant-free vacutainers for the isolation of serum, and in heparin-coated vacutainers for the experiments that required viable leukocytes [29]. Participant demographic information (age, sex, height) and health status (components of the Charlson Comorbidity Index [CCI], body mass index [BMI], medication history, vaccination history, and frailty) were provided at the time of sample collection. Based on the five Fried frailty criteria (weight loss, exhaustion, low physical activity, slowness, weakness), the participants were divided into three categories: non-frail (score 0), pre-frail (score 1–2) and frail (score 3–5). Only non-frail, healthy participants [as defined in Ref [30]]. who had not required antibiotics within two weeks of their single sample collection, were included in this analysis.

### Animals

All animal care and experiment protocols were approved by the McMaster Animal Research Ethics Board (AUP 21-04-13) and performed in accordance with the Canadian Council on Animal Care guidelines. Specific-pathogen-free male and female mice were maintained under a 12-h light-dark cycle at 22 ± 2 °C and 55 ± 5% air humidity at the McMaster Central Animal Facility (Hamilton, ON, Canada). To protect from age-related obesity, all mice were provided with an exercise wheel. Mice had *ad libitum* access to a 14% protein maintenance diet (Envigo Teklad Diets 2914, Madison, WI) and autoclaved reverse osmosis water. The C57BL/6J wildtype (WT) and B6.129s-*Tnf*^*tm1Gkl*^/J (TNF-α knockout; *Tnf*^*−/−*^) mice were obtained from The Jackson Laboratory (Bar Harbor, ME, USA) and bred in-house.

### Measurements of cellular and soluble inflammation

**Fig. 1:**
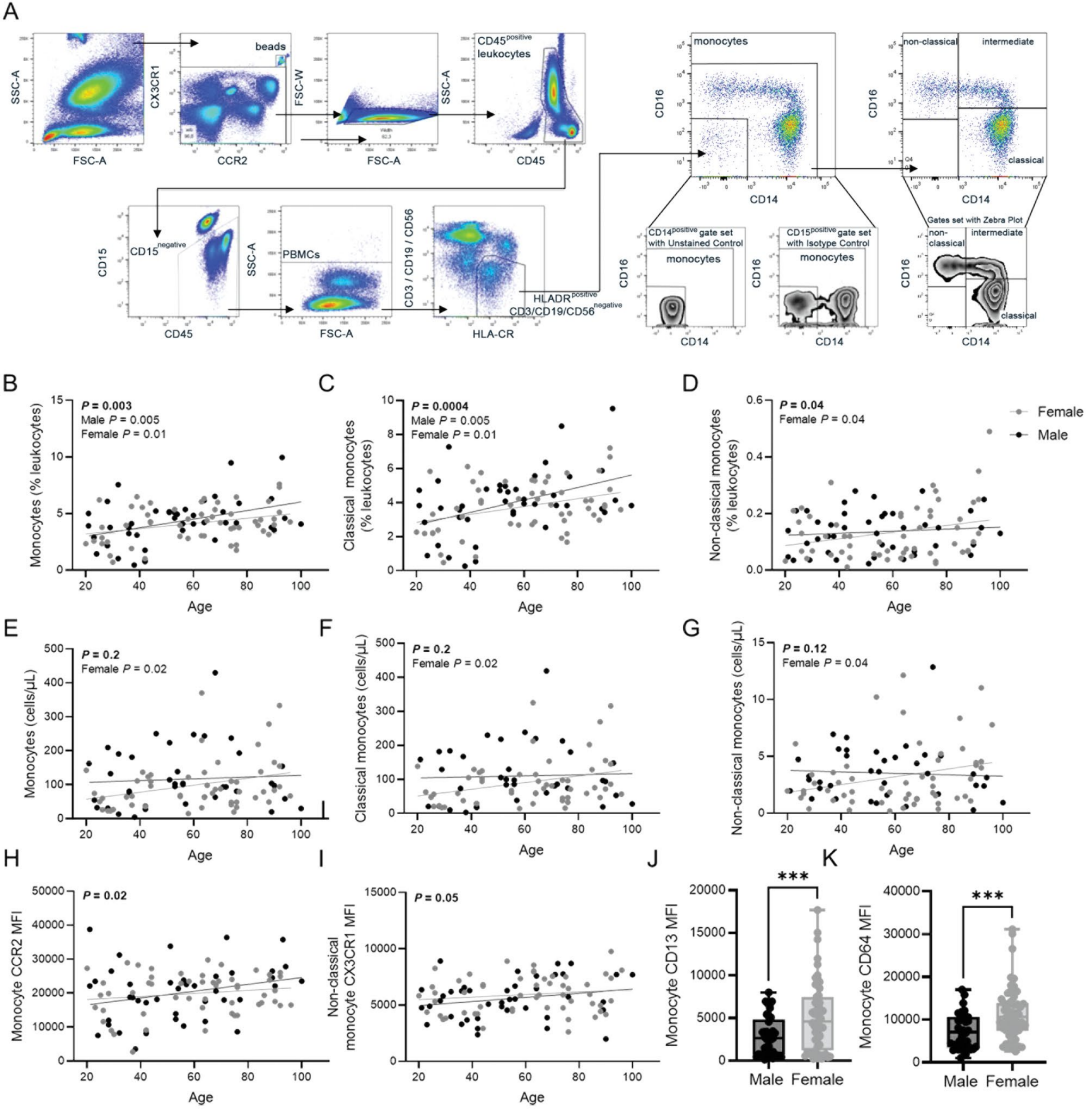
Peripheral blood monocyte populations change with age and sex. (**A**) The flow cytometry data gating strategy for peripheral whole-blood immunophenotyping of classical (CD14^+^CD16^−^), intermediate (CD14^+^CD16^++^) and non-classical (CD14^−^CD16^++^) monocyte populations. Both age and sex altered monocyte prevalence (as a proportion of total CD45^+^ leukocytes): total monocytes (**B**), classical monocytes (**C**), non-classical monocytes (**D**). Absolute monocyte numbers were not significantly affected by chronological age in the whole population (males and females) but showed a differential influence of biological sex. Total monocyte (**E**), classical monocyte (**F**), and non-classical monocyte (**G**) numbers increased in females with age. There were no sex differences in the surface expression of mobilization markers CC-chemokine receptor 2 (CCR2) and CX_3_CR_1_, though both increased with age in the whole population (**H**, **I**). In contrast, there was no age-associated change in the surface expression of monocyte activation markers CD13 and CD64 (data not shown), but expression was higher in females, inclusive of all ages (**J**, **K**). Monocyte surface receptor expression as mean fluorescence intensity (MFI). Data is shown as a dot for each participant. Subjects are color coded according to their biological sex (Male – black; female – grey). Statistical significance was assessed by simple linear regression (**A**-**I)** and Students’ *t* test (**J**,**K**)

In humans, circulating monocytes were quantified by multi-color flow cytometry as previously described [31, 32]. In brief, direct application of monoclonal antibodies (Additional File S1) to 100 µL whole blood was performed for 30 min at room temperature. Following staining, samples were fixed and red blood cells were lysed using 1 x Fix/Lyse Buffer (eBioscience, Thermo Fisher Scientific, Waltham, MA, USA) for 10 min. Samples were washed with PBS, resuspended in FACS Wash (5 mM EDTA, 0.5% BSA in PBS) and stored in the dark at 4ºC until analysis on a LSRII (BD Biosciences). Absolute numbers of monocyte populations were determined using CountBright™ absolute counting beads (Invitrogen Life Technologies, Carlsbad, CA, USA). The hierarchical gating strategy to determine circulating immune populations are shown in Fig. 1, set with appropriate isotype and unstained controls. The mouse tissues were processed and analyzed by flow cytometry based on our previous protocols [33]. Serum cytokines, chemokines and growth factors were quantified using human high sensitivity Discovery Assays (Eve Technologies). Measured cytokines included: granulocyte macrophage colony-stimulating factor (GM-CSF), interferon gamma (IFNγ), interleukin (IL)-1β, IL-2, IL-4, IL-5, IL-6, IL-8, IL-10, IL-12[p70], IL-13, IL-17 A, IL-23, TNF, vascular endothelial growth factor-A (VEGF-A), interferon gamma-induced protein 10 (IP-10; also known as C-X-C motif chemokine legend 10 [CXCL10]), and monocyte chemoattractant protein-1 (MCP-1). Measurements of C-reactive protein (CRP) and hCAP18/LL-37 were performed using human ELISA kits, following specifications of the manufacturer (ThermoFisher # KHA0031, #88-52103-22).

### Measurements of intestinal barrier function

Circulating muramyl dipeptide (MDP) and LPS in human sera was detected using a colourimetric reporter bioassay as previously described [10]. This assay quantifies NF-κB activation in response to the pattern recognition receptors nucleotide-binding oligomerization domain 2 (NOD2) and toll-like receptor (TLR)-4, respectively. Briefly, HEK293T cells were transfected with pNifty2-SEAP (Invivogen, CA, USA) and NOD2, generating an MDP-responsive reporter line. LPS-responsive reporter lines were created by stable transfection with pNifty-2-SEAP plasmid HEK293 cells expressing TLF-4, MD2 and CD14 (Invivogen, CA, USA). Cells were seeded at 40,000 cells per well in a 96-well plate in complete DMEM for 24 h. The media was removed and heat-inactivated serum (diluted 1:10) was added with HEK Blue Detection Media (Invivogen, CA, USA) to a final volume of 200 mL. Readings were performed at 650 nm, 24 h after stimulation and background levels were subtracted from relative absorbance units. Both assays were performed in triplicate. For detection of human intestinal fatty acid binding protein (I-FABP) and zonulin, commercially available ELISAs (ab193700 and ab219048, respectively) were performed on serum following manufacturers specifications.

In mice, intestinal permeability was determined by fluorescein isothiocyanate (FITC)-dextran assay as previously described [10]. Briefly, mice were fasted for 6 h then orally administered 150 µl of 3–5 kDA FITC-dextran (Sigma-Aldrich #46944, 80 mg/ml in PBS [pH 7.4]). Blood obtained from the tail vein 1 h prior to, and 4 h following gavage were sampled into tubes containing citrate-phosphate-dextrose solution (15% v/v). Plasma was obtained after centrifugation 5000 rpm for 10 min at 4 °C. Plasma was diluted 1:10 (*v*/*v*) in PBS (pH 7.4) and added to a 96-well microplate in duplicate. Fluorescence was measured spectrophotometrically with an excitation wavelength of 485 nm and an emission wavelength of 530 nm, with subtraction of background levels.

Trans-Epithelial Electrical Resistance (TEER) of co-cultured epithelial cells (Caco-2 [ATCC HTB-37]), goblet cells (HT-29-MTX-E12 [ATCC HTB-38], and Raji-B lymphocytes (ATCC CCL-86) was used to mimic the intestinal barrier as previously described [34]. To initiate cytokine induced changes in TEER, cytokine or vehicle control were introduced at various concentrations for 48 h at 37 °C, 5% CO2 and 90% relative humidity. TEER measurements were taken in duplicate for each well using a EVOM2 epithelial volt/ohm meter with chopstick electrodes (World Precision Instruments) at baseline and following treatment.

### Quantification of microbial metabolites

The short-chain fatty acids (SCFA) acetic acid, propionic acid, isobutyric acid, butyric acid, isovaleric acid, pentanoic acid and lactic acid were analyzed from human serum samples. Acidified samples were extracted twice with propyl formate, derivatized with N-Methyl-N- (tert-butyldimethylsilyl) trifluoroacetamide and analyzed by GCMS. For assessment of kynurenine, tryptophan and indole-3-acetic acid, serum samples were extracted with MeOH/H_2_0 (1:1) twice, and LCMS was performed on an Agilent 6550 Q-TOF using a Kinetex C18 column 2.6 μm (50 × 3.00 mm, 100Å). The flow rate was 200 µL/min. Calibration curves were used to obtain analyte concentration. Calibration curves for all analytes were linear over the range of interest, with R^2^ > 0.98.

### Statistical analysis

All statistical analysis were performed using R [35] version 4.1.2, GraphPad Prism version 9.2 (San Diego, CA, USA) and FlowJo™ (Version 10.8.1 Ashland, OR, USA). Data was tested for normality using a Shapiro-Wilk test. In instances where data failed the normality test it was natural log transformed. To compare age and sex differences among groups, a two-way analysis of variance (ANOVA) with Tukey’s multiple comparison test was used. A Mann-Whitney non-parametric test was used for comparing zonulin and I-FABP between sexes as these datasets failed normality tests even after several transformation attempts. Biological sex differences in comorbidities were determined using a Fisher’s exact test. To evaluate the association of biological sex with age-associated changes to monocyte populations in humans, we performed simple linear regressions. A Pearson correlation matrix was used to evaluate associations between immune parameters and markers of intestinal barrier function. To examine the temporal relationship between inflamm-aging and barrier function, a mediation analysis was used as recommended in Ref [36]., using the ‘Causal Mediation Analysis’ package in R [37]. Outliers in data were removed using the Grubbs’ method (α = 0.05).

## Results

### Demographics and comorbidities

The characteristics of the study population are displayed in Table 1. The participants ranged from 20 to 100 years of age and consisted of 54 females (56.8%) and 41 males. All participants fit the World Health Organization definition of healthy agers [30], and none were identified as frail based on the Fried frailty criteria [38]. In brief, healthy agers maintained their mental and physical capacity and did not suffer weight loss, exhaustion, low physical activity, slowness or weakness. Of the participants, 17.8% were young adults (20–35 years of age), 43.1% were adults (36–65 years of age), and 38.9% were older adults (> 65 years of age). There were no significant differences in body mass index (BMI) or sex distribution among age groups. Consistent with the rates in the Canadian population, females had greater incidence of hypothyroidism [39] and mood disorders [40].

**Table 1:** Summary of group characteristics and comorbidities

	Females						Males					
	Young adult (*n* = 9)		Middle aged (*n* = 21)		Older adult (*n* = 24)		Young adult (*n* = 8)		Middle aged (*n* = 20)		Older adult (*n* = 13)	
	Mean (SD)	Median (IQR)	Mean (SD)	Median (IQR)	Mean (SD)	Median (IQR)	Mean (SD)	Median (IQR)	Mean (SD)	Median (IQR)	Mean (SD)	Median (IQR)
Age (yrs)	27(4.5)	27 (25–29)	51.2(10.3)	53(42–63)	81.3(8.9)	83(74–88)	27.5(4.5)	28.5(24–31)	48.6(9.8)	51(39–56)	82(10.8)	83(72–89)
BMI (kg/m^2^)	22(3.0)	27(20–24)	25(3.4)	25.7(22–27)	26.2(3.8)	26(23–27)	24.8(5.4)	23.8(22–26)	26(4.3)	26(23–28)	25.4(2.9)	25(23–26)
	% Positive (n)											
CMV positive	11.1 (1)		47.6 (10)		70.8 (17)		12.5 (1)		25 (5)		76.9 (10)	
Smoker	0 (0)		9.5 (2)		12.5 (3)		12.5 (1)		10.0 (2)		23.1 (3)	
**Comorbidities**												
Hypertension	11.1 (1)		4.7 (1)		20.8 (5)		0 (0)		5.0 (1)		15.3 (2)	
Diabetes mellitus	0 (0)		0 (0)		4.2 (1)		0 (0)		5.0 (1)		15.3 (2)	
Cardiovascular	0 (0)		14.2 (3)		8.3 (2)		0 (0)		5.0 (1)		30.7 (4)	
Hypothyroidism**	33.3 (3)		4.7 (1)		16.6 (4)		0 (0)		0 (0)		0 (0)	
Mood*	33.3 (3)		14.2 (3)		12.5 (3)		12.5 (1)		0 (0)		0 (0)	
Joint	0 (0)		14.2 (3)		41.6 (10)		0 (0)		15.0 (3)		23.1 (3)	
GI	0 (0)		0 (0)		12.5 (3)		12.5 (1)		0 (0)		0 (0)	
GERD	0 (0)		4.7 (1)		4.2 (1)		0 (0)		15.0 (3)		7.7 (1)	
Respiratory	11.1 (1)		14.2 (3)		12.5 (3)		12.5 (1)		15.0 (3)		38.4 (5)	
Osteoporosis	0 (0)		4.7 (1)		12.5 (3)		0 (0)		0 (0)		0 (0)	
Other	0 (0)		4.7 (1)		8.3 (2)		0 (0)		10.0 (2)		7.1 (1)	
Cancer history	0 (0)		0 (0)		25 (6)		0 (0)		5.0 (1)		30.7 (4)	

Young adults (20–35 years of age); Middle aged (36–65 years of age); Older adult (> 65 years of age). Abbreviations: *BMI* body mass index, *GI* gastrointestinal, *CMV* cytomegalovirus, *GERD* gastroesophageal reflux disease, *SD* standard deviation, *IQR* interquartile range. Cardiovascular conditions included: atherosclerosis, tachycardia, hyperlipidemia, premature ventricular contractions, ventricular fibrillation, and heart disease. Respiratory conditions included: asthma, chronic bronchitis, interstitial lung disease, chronic obstructive pulmonary disorder. GI conditions included: gastrointestinal reflux disease, inflammatory bowel disease and irritable bowel syndrome. Joint comorbidities included: gout, rheumatoid arthritis, osteoarthritis, and psoriatic arthritis. The Hypothyroidism category includes goiters. Other comorbidities included: Parkinson’s disease, acrodermatitis continua of Hallopeau, hepatic encephalopathy and psoriasis. As detailed fully under the Methods section, BMI comparisons utilized a 2-way ANOVA test. Sex comparisons of comorbidities were performed using a Fisher’s exact test. *P < 0.05, **P < 0.01

### Biological sex differences in age-associated monocyte populations

Age-related changes in monocyte subset numbers have been previously reported; however, it is unclear whether biological sex impacts age-related differences in monocyte subset numbers or proportions. Using flow cytometry, we quantified monocyte subsets as well as their surface expression of migratory and activation markers. The flow cytometry gating strategy to identify monocytes is illustrated in Fig. 1A and results are summarized in Tables 2 and 3. We found an increase in the relative proportion (as a percentage of total CD45^+^ leukocytes) of total monocytes (Fig. 1B) and classical monocytes (Fig. 1C) in both sexes, whereas only females had an increase in the proportion of non-classical monocytes (Fig. 1D). Further analysis revealed an age-associated increase in the absolute numbers of total monocytes, classical monocytes and non-classical monocytes in females only (Fig. 1E-G), demonstrating sex-specific monocyte aging trajectories. There were no age or sex specific differences in intermediate monocyte subsets. Discrepancies between monocyte proportions and numbers could be attributed to a decrease in T cell numbers in males with age (Additional File S2).

Mobilization of monocyte populations into and out of the circulation occurs in response to chemokines. To understand the altered abundance of classical and non-classical monocytes in circulation, we investigated surface expression of CC-chemokine receptor 2 (CCR2) and CX_3_CR_1_. Both CCR2 and CX_3_CR_1_ increased with age on monocytes and non-classical monocytes, respectively (Fig. 1H, I); however, no sex-specific changes were observed. In contrast, there were no age-associated changes in the surface expression of monocyte activation markers CD13 and CD64, but females had higher expression of these markers when compared to males (Fig. 1J, K).

**Table 2:** Whole-blood myeloid cell prevalence by biological sex and chronological age

	Sex	Young adult (*n* = 8 M; 9 F)		Adult (*n* = 20 M; 21 F)		Older adult (*n* = 13 M; 24 F)	
		Mean (SD)	Median (IQR)	Mean (SD)	Median (IQR)	Mean (SD)	Median (IQR)
**% CD45** ^**+**^ **leukocytes**							
Monocytes	Male	3.7(2.16)	3.77(1.8–5.5)	3.8(1.6)	4.11(3.1-5.0)	5.4(2.2)	4.58(3.9–6.4)
	Female	2.8(1.5)	2.5(2-3.8)	4.36(1.51)	4.5(3-5.6)	4.4(1.5)	4.2(3.6–5.3)
Classical monocytes	Male	3.4(2.1)	3.6(1.17–4.99)	3.49(1.56)	3.9(3-4.7)	5.02(1.98)	4.1(3.7–6.1)
	Female	2.5(1.49)	2.23(1.8–3.4)	3.96(1.4)	4.15(2.7–5.2)	4.11(1.4)	3.8(3.2–5.1)
Intermediate monocytes	Male	0.15(0.16)	0.10(0.03–0.27)	0.17(0.13)	0.15(0.06–0.25)	0.19(0.12)	0.18(0.07–0.25)
	Female	0.16(0.12)	0.15(0.06–0.23)	0.18(0.2)	0.12(0.06–0.21)	0.15(0.11)	0.11(0.09–0.16)
Non-classical monocytes	Male	0.11(0.07)	0.11(0.04–0.19)	0.14(0.07)	0.15(0.07–0.2)	0.14(0.08)	0.14(0.07–0.19)
	Female	0.12(0.07)	0.12(0.06–0.19)	0.10(0.07)	0.07(0.05–0.12)	0.16(0.11)	0.15(0.06–0.24)
**% Monocytes**							
Classical monocytes	Male	86.1(14.3)	94.4(71–97)	87.9(11.1)	92.1(86–95)	93.1(3.2)	93.3(90–96)
	Female	86.4(10.2)	89.9(76–92)	92.6(5.0)	94.2(88–96)	92.05(4.47)	92.8(89–94)
Intermediate monocytes	Male	6.0(7.6)	2.2(0.79–10.6)	5.22(3.6)	4.2(1.7–8.5)	3.4(1.9)	3.0(1.9–4.4)
	Female	7.3(6.2)	5.4(2.6–10.5)	4.4(3.4)	3.8(1.6–6.5)	3.6(2.7)	2.9(1.9–3.5)
Non-classical monocytes	Male	5.5(6.3)	1.5(1.2–12.1)	5.3(6.4)	3.2(1.9–5.1)	3.0(1.6)	2.6(1.8–4.6)
	Female	4.5(3.1)	3.1(2.1–7.9)	2.6(2.4)	1.8(1.3–3.1)	3.7(2.4)	3.5(1.9–4.7)
**Correlations by chronological age**							
**Variables**			**Beta**	**Std. Error**	**R** ^**2**^	***P***	
**% CD45** ^**+**^ **leukocytes**							
Monocytes	Male		0.038	0.01	0.17	0.006*	
	Female		0.02	0.009	0.10	0.01*	
Classical monocytes	Male		0.036	0.012	0.172	0.006*	
	Female		0.023	0.005	0.11	0.01*	
Intermediate monocytes	Male		0.001	0.0009	0.033	0.24	
	Female		0.0001	0.0009	0.0007	0.84	
Non-classical monocytes	Male		0.000	0.000	0.01	0.49	
	Female		0.001	0.0005	0.07	0.04*	
**% Monocytes**							
Classical monocytes	Male		0.14	0.07	0.09	0.05*	
	Female		0.05	0.03	0.04	0.14	
Intermediate monocytes	Male		-0.03	0.03	0.04	0.20	
	Female		-0.04	0.02	0.06	0.06	
Non-classical monocytes	Male		-0.05	0.03	0.06	0.11	
	Female		-0.0007	0.01	0.00	0.96	

Top: Summary statistics of peripheral blood monocyte cell proportions in young adults (20–35 years of age), adults (36–64 years of age) and older adult (≥ 65 years of age). Bottom: Results of simple linear regression test showing how monocyte prevalence change with chronological age in males and females

**Table 3:** Whole-blood myeloid cell numbers^a^ by biological sex and chronological age

	Sex	Young adult (*n* = 8 M; 9 F)		Adult (*n* = 20 M; 21 F)		Older adult (*n* = 13 M; 24 F)	
		Mean (SD)	Median (IQR)	Mean (SD)	Median (IQR)	Mean (SD)	Median (IQR)
**Cell population**							
Monocytes	Male	103.1(79.2)	93.2(31–183)	113.7(79.6)	100.5(42–180)	127.3(109.)	93.6(60–137)
	Female	54.3(38.4)	50.3(24–68)	107.8(81)	98.1(62–129)	109.3(77.6)	84.9(52–150)
Classical monocytes	Male	100.5(80.29)	128.8(20–181)	106.2(78.0)	91.5(35–179)	119.2(106)	85.6(56–164)
	Female	48.9(38.4)	47.2(19–60)	98.1(72.9)	94.3(37–119)	101.6(74.0)	77.9(49–140)
Intermediate monocytes	Male	3.6(2.8)	2.8(1-6.9)	4.8(3.8)	3.7(2.8–6.5)	4.0(3.1)	3.9(1.1–6.2)
	Female	2.7(1.7)	2.0(1.6–4.3)	5.4(6.5)	3.1(1.9–5.9)	3.4(2.4)	2.6(1.4–5.4)
Non-classical monocytes	Male	2.7(1.1)	2.7(1.9–3.2)	3.8(2.1)	4.1(1.5–5.6)	3.5(3.2)	3.1(1.5–4.7)
	Female	2.3(1.6)	2.0(1.5-3.0)	3.1(3.4)	1.9(1.0-3.4)	3.7(2.7)	3.1(1.3-5.0)
**Correlations by chronological age**							
**Variables**			**Beta**	**Std. Error**	**R** ^**2**^	***P***	
Monocytes	Male		0.25	0.64	0.004	0.69	
	Female		1.03	0.45	0.09	0.02*	
Classical monocytes	Male		0.16	0.66	0.001	0.8	
	Female		0.98	0.42	0.09	0.02*	
Intermediate monocytes	Male		-0.003	0.02	0.001	0.90	
	Female		-0.004	0.02	0.001	0.86	
Non-classical monocytes	Male		-0.01	0.01	0.003	0.72	
	Female		0.03	0.02	0.07	0.048*	

^a^ All cell numbers are cells/µL. Top: Summary statistics of peripheral blood myeloid cell numbers in young adults (20–35 years of age), adults (36–65 years of age) and older adult (> 65 years of age). Bottom: Results of simple linear regression test showing how myeloid cell numbers change with chronological age in males and females

### Inflamm-aging is more pronounced in females

**Fig. 2:**
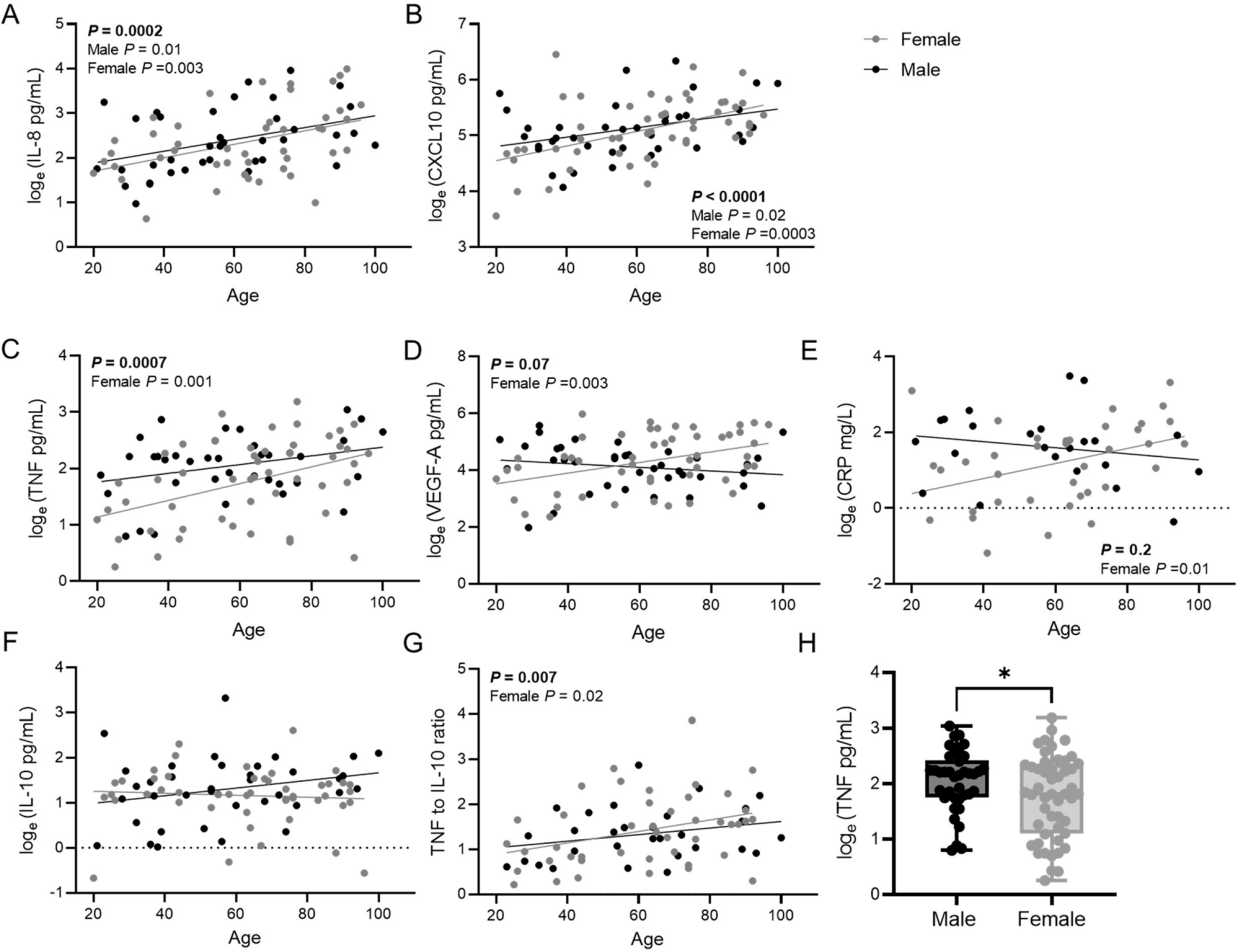
Age-associated inflammation is altered by biological sex. Simple linear regression showing a significant age-associated increase in the chemokines (**A**) IL-8 and (**B**) CXCL10 in both sexes. Females had an increase in circulating (**C**) TNF, (**D**) VEGF-A and (**E**) CRP with age. (**F**) Although no changes in IL-10 occurred, (**G**) there was an increase in the TNF to IL-10 ratio in females with age. (**H**) Males had higher overall TNF levels, but unlike in the females, these levels did not change with age. Statistical significance was assessed by a Student’s *t* test. *P ≤ 0.05

To assess whether immune cell activation or inflammatory state differ by biological sex, we quantified peripheral blood cytokines and chemokines. Consistent with previously published data, levels of circulating chemokines, such as IL-8 and CXCL10 increased with age in study participants (Fig. 2A, B). The increase in these inflammatory mediators was evident in both sexes; however, there was a larger impact of age in females. Circulating TNF, VEGF-A and CRP likewise increased with chronological age in females, but not males although there was a trend (Fig. 2C-E). We did not see any age-specific changes in other major inflammatory cytokines including IFNγ, IL-1β, IL-2, IL-4, IL-5, IL-8, IL-12[p70], IL-13, IL-17 A, and IL-23, though there was a tendency toward an increase in IL-6 in males with age (Additional File S3). The anti-inflammatory cytokine, IL-10, also did not change with age; however, the ratio of TNF to IL-10 significantly increased with age in females but not males (Fig. 2F, G), demonstrating that the balance of pro-inflammatory and anti-inflammatory cytokines change in a sex dependent manner with age. However, we should note that despite females having more pronounced age-associated inflammation, males had higher absolute TNF in peripheral blood that did not change with age (Fig. 2H).

### Females have lower barrier integrity

Accumulating evidence in mice suggests that intestinal barrier dysfunction may be a driving factor of inflamm-aging [reviewed in Ref [41]]. We investigated serum markers of barrier function to determine if there was a relationship between barrier function and inflamm-aging in humans. Circulating indicators of epithelial permeability/damage (zonulin [42, 43], I-FABP [44]), microbiota-derived products (LPS, MDP) and metabolites (SCFAs [acetic acid, propionic acid, isobutyric acid, butyric acid, isovaleric acid, pentanoic acid and lactic acid] and derivatives of the kynurenine pathway [45] [kynurenine, tryptophan and indole-3-acetic acid]), were used as non-invasive markers of intestinal barrier function. Dietary tryptophan can be oxidized, via the kynurenine pathway, into kynurenine in the liver of the host [46]; however, accumulating evidence highlights a crucial role of the gut microbiota in determining tryptophan availability for the host by balancing the microbial tryptophan metabolism in the gut [47]. As such, we included the kynurenine-tryptophan metabolites as microbial markers but acknowledge they may be host-derived as well. The human antimicrobial host defense peptide hCAP18/LL-37 was also included as a biomarker of epithelial barrier function, based on studies showing it contributes to barrier integrity in mice [48]. hCAP18/LL-37 is the sole human cathelicidin peptide, orthologous to murine mCRAMP. Cathelicidin is expressed by intestinal epithelial cells as well as resident immune cells in the intestine including neutrophils, monocytes and macrophages in humans and in mice. In addition to direct antimicrobial and immunomodulatory properties, cathelicidin has been shown to stimulate re-epithelialization [49] and increase tight junction proteins [50] which are essential for intestinal barrier integrity. Intestinal cathelicidin also maintains the balance between pro- and anti-inflammatory factors [50] and protects against colonization with epithelial adherent bacterial pathogens [51]. Not surprisingly, cathelicidin deficiency in mice results in pathological dysregulation of barrier function and immune environment with increased susceptibility to gastrointestinal infection, DSS-induced colitis and gluten-induced enteropathy [48–51], all ameliorated by restoration of intestinal cathelicidin (e.g. by treating with a cathelicidin-secreting strain of *Lactococcus lactis*) [52, 53].

**Fig. 3:**
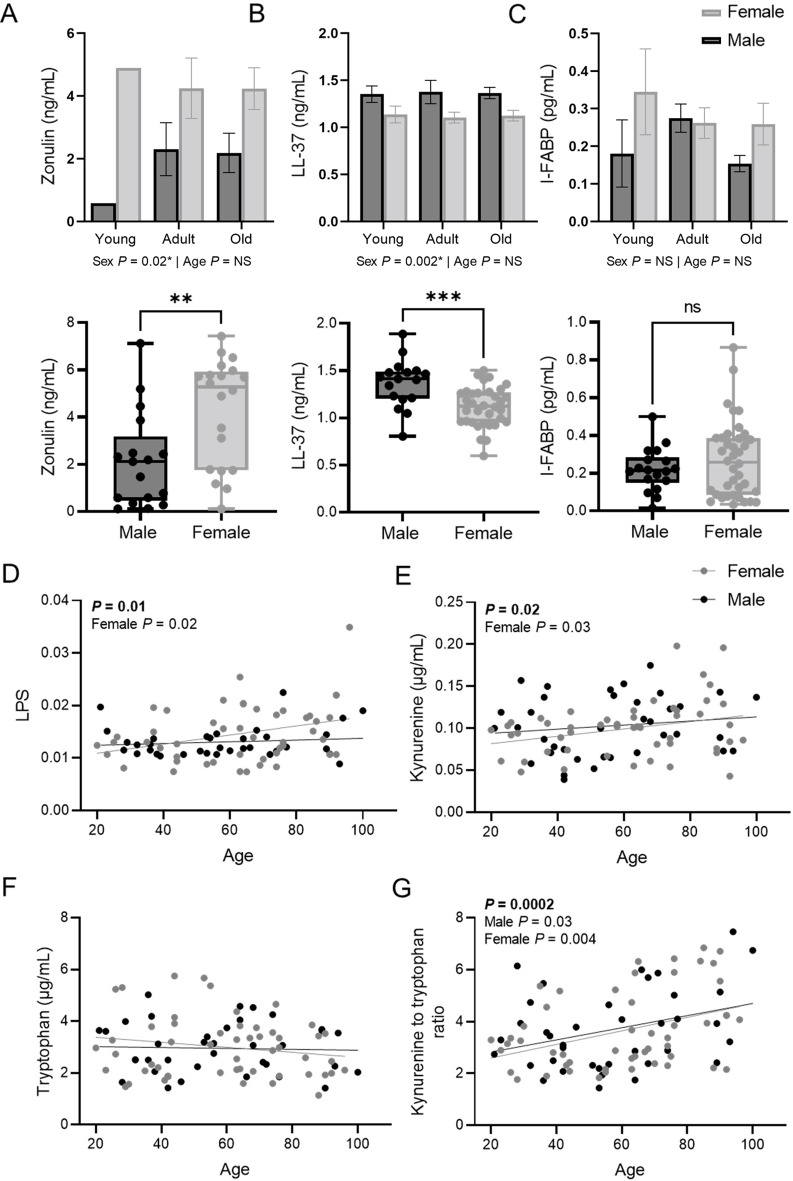
Females have lower intestinal barrier integrity. Noninvasive biomarkers of intestinal barrier function showed (**A**) higher circulating zonulin in females, indicative of increased intestinal barrier permeability. No age-associated changes in zonulin were observed when categorized as young adults (20–35 years of age), adults (36–65 years of age), and older adults (> 65 years of age) (**B**) Assessment of the human host defense peptide hCAP18/LL-37 showed lower circulating hCAP18/LL-37 in females as compared to males, which was age independent. (**C**) There were no age or sex differences in circulating I-FABP. (**D**) Circulating LPS increased with age, driven by an increase in females only. (**E**) Circulating kynurenine increased with age in females. (**F**) There were no age- or sex-associated changes in circulating tryptophan levels. (**G**) The ratio of kynurenine to tryptophan increased with age in both biological sexes. Statistical significance was assessed by a two-way ANOVA (A-C top), Student’s *t* test (**A**-**C** bottom) and simple linear regression (**D**-**G**). Data is shown as a dot for each participant. Subjects are color coded according to their biological sex. **P ≤ 0.01

The results showed an impact of sex, but not age, on circulating zonulin levels whereby females had higher zonulin expression (Fig. 3A), which may be indicative of increased intestinal permeability. This was accompanied by lower circulating hCAP18/LL-37 in females across the life-course (Fig. 3B), with no differences in young or old participants. No age or sex differences in I-FABP were observed (Fig. 3C). In agreement with previous reports [54], we found an increase in circulating LPS with age. When we considered variation by biological sex, we found that the apparent age-associated increase in circulating LPS was predominantly driven by increases in females (Fig. 3D). There were no associations between MDP, age or sex. The integrity of the intestinal epithelium is maintained, in part, by bacterial metabolites such as SCFAs. An analysis of circulating SCFAs revealed no age- or sex-driven differences in SCFAs (Additional File S4). Likewise, there were no age or sex-dependent effects on circulating tryptophan or indole-3-aa; however, circulating kynurenine increased with age, resulting in a higher kynurenine to tryptophan [KT] ratio (Fig. 3E-G). When we assessed kynurenine by biological sex we found a female-specific increase in the levels of kynurenine with age. Though not significant, males had a similar trend. Overall, this data shows that females have higher indicators of compromised barrier integrity than males, establishing that there is a strong effect of biological sex, independent of chronological age, on intestinal barrier integrity.

### Impaired barrier function correlates with the activation and recruitment of monocytes

**Fig. 4:**
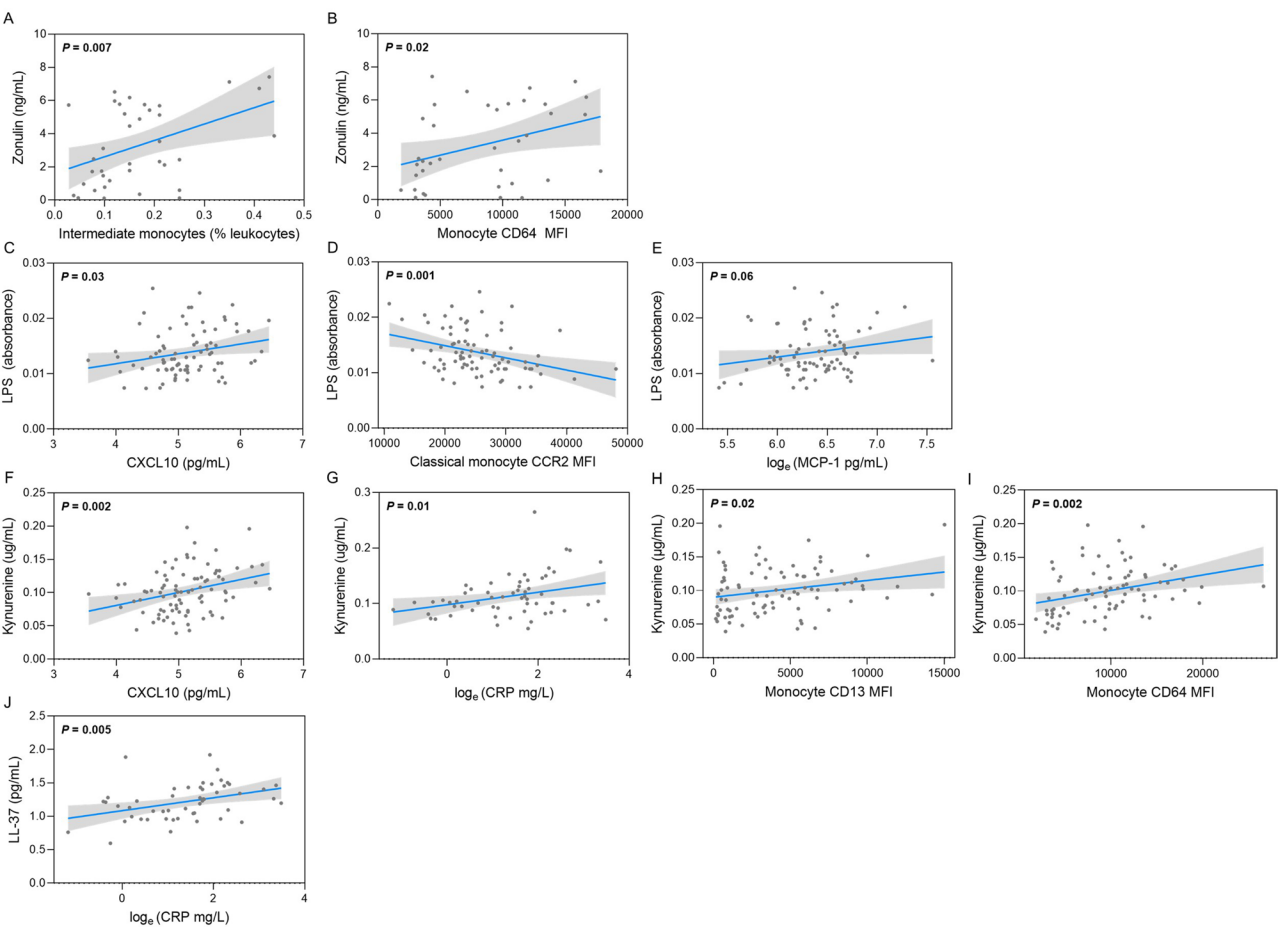
Associations between circulating markers of intestinal barrier disfunction and immune parameters. Pearson correlation analysis showing a correlation between circulating zonulin and (**A**) intermediate monocyte proportions and (**B**) monocyte expression of CD64. Peripheral blood LPS was significantly correlated with circulating (**C**) CXCL10, (**D**) classical monocytes expressing CCR2 and (**E**) MCP-1. The bacterial metabolite, kynurenine, was positively associated with circulating (**F**) CXCL10, (**G**) CRP, (H) monocyte expression of CD13 and (**I**) monocyte expression of CD64. (**J**) The human antimicrobial host defense peptide, hCAP18/LL-37 was positively associated with CRP. Data is shown as a dot for each participant. Shaded area represents 95% confidence intervals

We next sought to determine if the differences in barrier integrity correlated with soluble and cellular markers of inflammation. Results from the Pearson correlation analyses revealed a positive association between circulating zonulin levels and the relative frequency of intermediate monocytes, as a proportion ofCD45^+^ leukocytes (Fig. 4A). Zonulin also had a positive association with CD64 expression on monocytes (Fig. 4B), which can lead to monocyte activation and further production of inflammatory cytokines [55]. Circulating LPS was correlated with the inflammatory chemokine, CXCL10 (Fig. 4C), but had an inverse relationship with classical monocytes expressing CCR2 (Fig. 4D). Surface expression of CCR2 on the total monocyte population likewise had a negative correlation with LPS. Monocytes expressing high CCR2 are the first to leave the bone marrow. Once they have entered circulation, they are recruited to sites of acute inflammation in response to CCL2/monocyte chemoattractant protein-1 (MCP-1) or differentiate into intermediate monocytes. We speculated that the inverse relationship between classical monocytes expressing CCR2 and the circulating LPS levels was a result of cells expressing the highest levels of CCR2 emigrating from the circulation. In support of this, we found a marginal (*P* = 0.06) correlation between circulating MCP-1 and LPS levels (Fig. 3E). Circulating kynurenine was positively associated with CXCL10 (Fig. 4F) and CRP (Fig. 4G). Levels of kynurenine in circulation also had a positive association with monocyte activation markers including CD13 and CD64 (Fig. 4H, I). Finally, we found that circulating hCAP18/LL-37 levels were positively associated with circulating CRP (Fig. 4J). Overall, these data demonstrate that impaired intestinal barrier integrity corresponds with higher inflammatory mediators.

### Inflammation as a driver of gut permeability

To further examine the associations between our observations of the effects of age on peripheral monocytes, soluble inflammatory mediators, intestinal barrier functions, and their modification by biological sex, we used a path analysis. Path analysis evaluates the extent to which the relationship between two variables can be explained by a third variable (the mediator). Although path analysis based on cross-sectional data cannot prove causality, it can strengthen the case for a causal relationship between exposure and outcome [36].

**Fig. 5:**
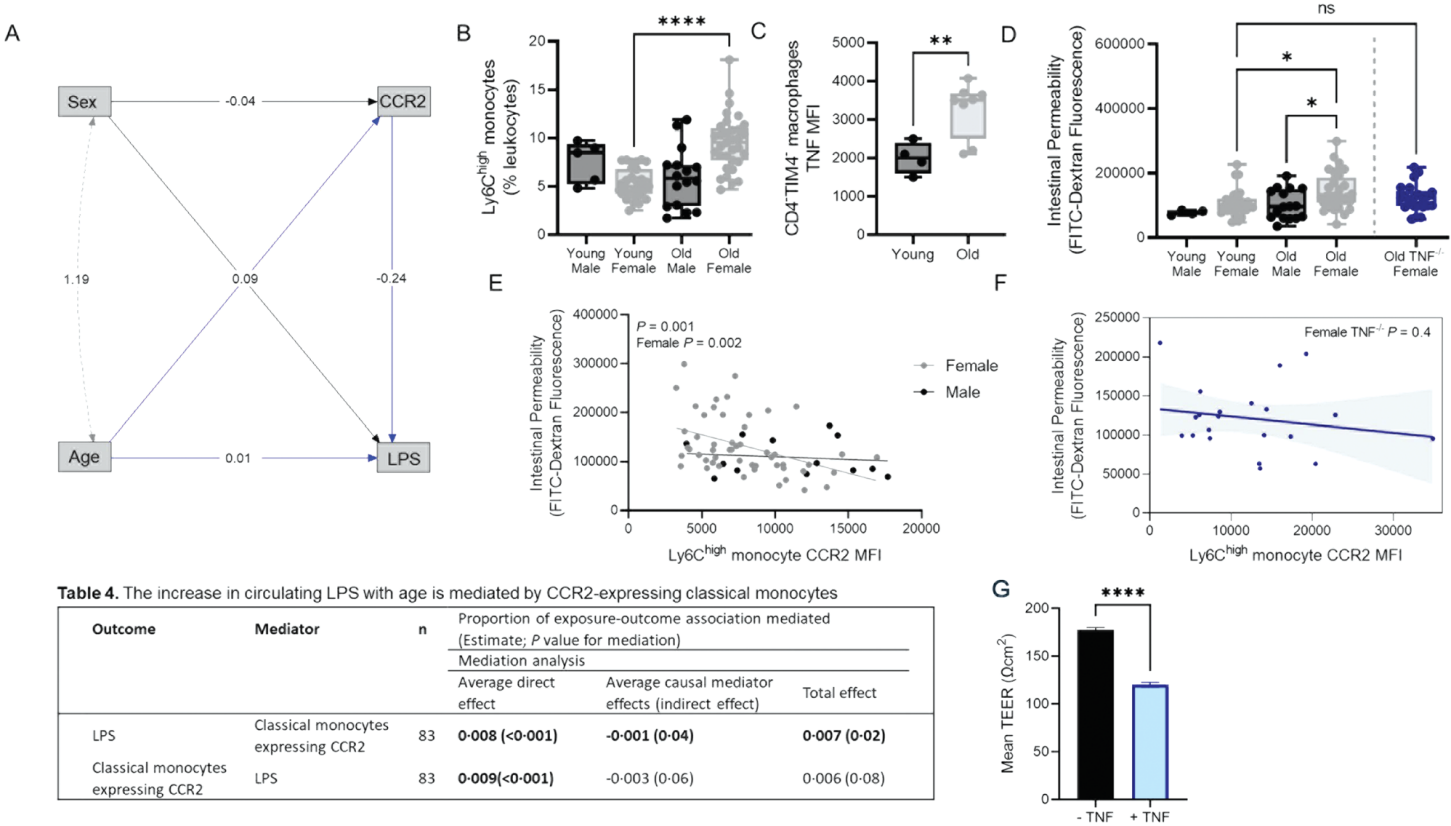
Intestinal barrier integrity is mediated by CCR2-expressing inflammatory monocytes in humans and mice. (**A**) Path analysis demonstrates that chronological age has a direct effect on circulating LPS levels and CCR2-expressing classical monocytes(CD14^+^CD16^−^) in humans. The amount of LPS in circulation is mediated by the monocytes in peripheral blood wherein the CCR2-expressing monocytes in circulation decreased with an increasing amount of circulating LPS. Effect estimates from the 100 simulations are shown in Table 4. In mice, (**B**)Ly6C^high^ monocyte prevalence (as a proportion of total CD45^+^ leukocytes) increased in the circulation of old wild-type females, but not old males. (**C**) Monocyte-derived CD4^−^TIM4^−^ colon macrophages have increased TNF expression in old mice. (**D**) Intestinal permeability, as measured by circulating FITC-dextran levels, increased in old wild-type females, but not old males or old *Tnf*^*−/−*^ females who are missing age-associated inflammation. (**E**) There was an inverse relationship between CCR2-expressing Ly6C^high^ monocyte and FITC-dextran in the circulation of females, but not males or (**F**) *Tnf*^*−/−*^ females. (**G**) Trans-epithelial electrical resistance (TEER) prior to- and following- administration of 2pg/mL TNF showed reduced barrier integrity following cytokine challenge

Herein, we evaluated whether the association between age and sex (exposures) and impaired barrier function (outcome) was mediated by inflammation (mediator) or vice versa. We did independent mediation analysis on five separate markers of barrier dysfunction (zonulin, LPS, kynurenine, KT and hCAP18/LL-37) and eight markers of immune function (TNF, inflamm-aging [TNF, CRP, IL-6], CXCL10, CRP, monocyte numbers, monocyte expression of CD13 and CD64 and classical monocytes expression of CCR2). These parameters were selected as they were the most likely to have an effect be mediated (i.e., they changed with age or sex and there was a correlation between immune and intestinal parameters). Of all the simulations, the only combination to have a mediated effect was between classical monocytes expressing CCR2 and LPS with age. We found the amount of LPS in circulation was mediated by CCR2-expressing classical monocytes wherein LPS in circulation increases as CCR2-expressing classical monocytes decrease (Fig. 5A). The reversal, LPS mediating an increase in CCR2-expressing classical monocytes, was not significant. This data indicates that changes in monocyte phenotype mediates changes in barrier integrity with age.

These findings were then considered in the context of mouse models. We hypothesized the most likely cause of enhanced gut permeability in old mice to be inflammation derived from immigrating CCR2-expressing Ly6C^high^ monocytes (the mouse equivalent to classical monocytes[CD14^+^CD16^−^] in humans) [56]. Flow analyses of peripheral blood collected from young (5–7 mo) and old (> 18 mo) wild-type (WT) C57Bl/6 mice revealed an increase in the prevalence of Ly6C^high^ monocytes in old female but not old male mice (Fig. 5B), compatible with our human data. Differentiation of theseLy6C^high^ monocytes into intestinal macrophages could change the composition, phenotype, and function of intestinal macrophages. Both human and mouse intestinal tissues contain macrophages derived from circulating monocytes and tissue-resident self-renewing populations [24, 57]. As we previously observed that increase age-associated paracellular permeability was localized to the colon [10], we examined colon monocyte-derived macrophage populations for evidence of increased inflammation. We found thatCD4^−^TIM4^−^ monocyte-derived macrophages in old mice had increased intracellular expression of TNF (Fig. 5C). Using a non-terminal gavage of 3–5 kDa FITC-dextran and measuring translocation of the FITC-dextran from the gut to the serum, we found that old female mice had a significant increase in intestinal permeability when compared to young mice and age-matched males (Fig. 5D). To determine if the differences in barrier permeability correlated with the changes in inflammatory monocytes, as we observed in humans, we performed a Pearson correlation analysis between CCR2-expressing Ly6C^high^ monocytes and intestinal permeability (i.e. FITC-dextran). In agreement with the human data, the results show a significant negative correlation between circulating CCR2-expressing Ly6C^high^ monocytes and intestinal permeability in females but not males (Fig. 5E). Thus, we reason that emigration of CCR2-expressing Ly6C^high^ monocytes from circulation and into the colon, increases inflammation in the local environment that reduces barrier integrity. Consistent with this, we found that that old female *Tnf*^*−/−*^ mice that are missing age-associated inflammation, do not have increased intestinal permeability, and the relationship between CCR2-expressing Ly6C^high^ monocytes and intestinal permeability is lost (Fig. 5D, F). We confirmed that TNF reduces barrier function using TEER, consistent with previous reports [58] (Fig. 5G). These findings in mice support the interpretation from the human data that immune remodeling resulting in inflamm-aging exacerbate barrier dysfunction in females with age.

## Discussion

Accumulating evidence suggests that inflamm-aging may underlie the pathogenesis of aging conditions, but whether biological sex contributes to the variations in cellular immune populations or soluble inflammatory factors that guide inflamm-aging, remains unclear. Herein, we reported age- and sex-specific differences in monocyte subsets and inflammatory mediators in the circulation of healthy individuals aged 20 to 100 years. We also considered whether age-associated increases in inflammatory profiles were mediated by impaired intestinal barrier function or vice versa. In contrast to the prevailing theory developed in mouse models [7, 8], the results show that impaired barrier function resulting in circulating LPS may not be the mediator of inflamm-aging. Rather, impaired barrier function is likely a consequence of, or is exacerbated by, the low-grade inflammation that increases with chronological age. The results demonstrate that females are more likely to experience inflamm-aging, which, when combined with their higher basal intestinal permeability, makes females more likely to accrue peripheral blood LPS and kynurenine with age. The sex-specific differences in inflammatory markers with age may be attributed to hormonal differences, environmental influences, or lifestyle factors, which warrant further investigation.

We show that age has a more pronounced impact on monocyte prevalence, phenotype, and function in females. In agreement with previous literature [59], there was an age-associated increase in the relative proportion of monocytes as a percentage of total leukocytes in both sexes. However, only females had a significant increase in total monocyte numbers, driven by an increase in both classical and non-classical monocyte subsets. We show that the increase in peripheral blood monocytes in females was accompanied by marked changes in inflammatory profiles. Although both sexes had increased peripheral blood chemokines with age (e.g. IL-8 and CXCL10), only females had increased TNF and CRP, indicative of inflamm-aging.

Earlier studies have suggested that inflamm-aging may be a consequence of sub-clinically elevated levels of circulating LPS [60]. The potential source of circulating LPS may be derived from compromised intestinal barriers or infections. In this study, we aimed to eliminate infections as a source of LPS by excluding participants who required antibiotics within two weeks of sample collection. Investigation of intestinal barrier integrity revealed sex-differences in non-invasive measures of barrier function. Females had consistently higher zonulin and lower hCAP18/LL-37 detected in the circulation, as compared to males. These observations indicate that females have reduced barrier integrity [49, 50] and may be less able to balance pro- and anti-inflammatory responses in the intestine [50]. Surprisingly, the increased permeability did not result in the passage of luminal contents such as LPS into the bloodstream in young adults, and there was no evidence that higher basal permeability in females triggered inflamm-aging. In contrast, we show that inflamm-aging was most likely the initial cause of enhanced bacterial products in circulation of females.

In support of this, we show that the increase in circulating LPS and kynurenine in females was robustly associated with aging and correlated with age-associated changes in chemokines, pro-inflammatory cytokines, and inflammatory status (i.e. CRP). These data are in agreement with previous studies, which have shown that inflammatory mediators shunt tryptophan metabolism towards its catabolite kynurenine, and that an increase in the kynurenine to tryptophan ratio in blood is associated with aging in humans [detailed in Ref [45]]. Additional research to elucidate the primary source of kynurenine (host vs. microbe) is needed in order to foster future interventions to restore KT homeostasis. Finally, we found an inverse relationship between CCR2-rich classical monocytes and LPS in circulation. We speculate that the inverse relationship between monocytes expressing CCR2 and the circulation of LPS levels is a result of cells expressing the highest levels of CCR2 emigrating from the circulation, but this would need to be experimentally determined. Using a mediation analysis, we demonstrated that the changes in circulating CCR2-rich classical monocytes mediated the increase in LPS with age, not vice versa. Collectively, this data demonstrates that age-associated inflammation and monocyte migration may be responsible for the increase in bacterial LPS and kynurenine in peripheral blood with age.

## Conclusion

Taken together, our results suggest that higher basal intestinal permeability in combination with age-associated inflammation, increases circulating bacterial products in females. Thus, targeting barrier function may slow the progression of inflamm-aging, but is unlikely to prevent it, whereas targeting inflamm-aging is likely to reduce circulating endotoxin.

## Electronic supplementary material

Below is the link to the electronic supplementary material.

Supplementary Material 1

Supplementary Material 2

Supplementary Material 3

Supplementary Material 4

## Data Availability

Data is publicly available at https://osf.io/bpz23/.

## Abbreviations

CRP: C-reactive protein
FITC-dextran: Fluorescein Isothiocyanate-Dextran
LPS: Lipopolysaccharide
TNF: Tumor necrosis factor-α
KT: Kynurenine to tryptophan ratio

## References

[1] Ferrucci L, Fabbri E. Inflammageing: chronic inflammation in ageing, cardiovascular disease, and frailty. Nat Rev Cardiol. 2018;15(9):505–22. 30065258 10.1038/s41569-018-0064-2 PMC6146930

[2] Fulop T, Larbi A, Dupuis G, Le Page A, Frost EH, Cohen AA, et al. Immunosenescence and Inflamm-Aging as two sides of the same Coin: friends or foes? Front Immunol. 2017;8:1960. 29375577 10.3389/fimmu.2017.01960 PMC5767595

[3] Franceschi C. Inflammaging as a major characteristic of old people: can it be prevented or cured? Nutr Rev. 2007;65(12 Pt 2):S173–6. 18240544 10.1111/j.1753-4887.2007.tb00358.x

[4] Franceschi C, Bonafe M, Valensin S, Olivieri F, De Luca M, Ottaviani E, et al. Inflamm-aging. An evolutionary perspective on immunosenescence. Ann N Y Acad Sci. 2000;908:244–54. 10911963 10.1111/j.1749-6632.2000.tb06651.x

[5] Harris TB, Ferrucci L, Tracy RP, Corti MC, Wacholder S, Ettinger WH Jr., et al. Associations of elevated interleukin-6 and C-reactive protein levels with mortality in the elderly. Am J Med. 1999;106(5):506–12. 10335721 10.1016/s0002-9343(99)00066-2

[6] Metchnikoff E. The prolongation of life. Putnam. 1908.

[7] Biagi E, Candela M, Fairweather-Tait S, Franceschi C, Brigidi P. Aging of the human metaorganism: the microbial counterpart. Age (Dordr). 2012;34(1):247–67. 21347607 10.1007/s11357-011-9217-5 PMC3260362

[8] Franceschi C, Campisi J. Chronic inflammation (inflammaging) and its potential contribution to age-associated diseases. J Gerontol Biol Sci Med Sci. 2014;69(Suppl 1):S4–9. 10.1093/gerona/glu057 24833586

[9] Mitchell EL, Davis AT, Brass K, Dendinger M, Barner R, Gharaibeh R, et al. Reduced intestinal motility, mucosal barrier function, and inflammation in aged monkeys. J Nutr Health Aging. 2017;21(4):354–61. 28346561 10.1007/s12603-016-0725-y PMC6057140

[10] Thevaranjan N, Puchta A, Schulz C, Naidoo A, Szamosi JC, Verschoor CP, et al. Age-Associated Microbial Dysbiosis promotes intestinal permeability, systemic inflammation, and macrophage dysfunction. Cell Host Microbe. 2017;21(4):455–66. e4. 28407483 10.1016/j.chom.2017.03.002 PMC5392495

[11] Walker EM, Slisarenko N, Gerrets GL, Kissinger PJ, Didier ES, Kuroda MJ, et al. Inflammaging phenotype in rhesus macaques is associated with a decline in epithelial barrier-protective functions and increased pro-inflammatory function in CD161-expressing cells. Geroscience. 2019;41(6):739–57. 31713098 10.1007/s11357-019-00099-7 PMC6925095

[12] Rahtes A, Li L. Polarization of low-Grade Inflammatory monocytes through TRAM-Mediated Up-Regulation of Keap1 by Super-low Dose Endotoxin. Front Immunol. 2020;11:1478. 32765513 10.3389/fimmu.2020.01478 PMC7378438

[13] Soeters PB, Luyer MD, Greve JW, Buurman WA. The significance of bowel permeability. Curr Opin Clin Nutr Metab Care. 2007;10(5):632–8. 17693749 10.1097/MCO.0b013e3282a0780e

[14] Rizzetto L, Fava F, Tuohy KM, Selmi C. Connecting the immune system, systemic chronic inflammation and the gut microbiome: the role of sex. J Autoimmun. 2018;92:12–34. 29861127 10.1016/j.jaut.2018.05.008

[15] Capaldo CT, Nusrat A. Cytokine regulation of tight junctions. Biochim Biophys Acta. 2009;1788(4):864–71. 18952050 10.1016/j.bbamem.2008.08.027 PMC2699410

[16] Marchiando AM, Shen L, Graham WV, Weber CR, Schwarz BT, Austin JR 2, et al. Caveolin-1-dependent occludin endocytosis is required for TNF-induced tight junction regulation in vivo. J Cell Biol. 2010;189(1):111–26. 20351069 10.1083/jcb.200902153 PMC2854371

[17] Franceschi C, Garagnani P, Parini P, Giuliani C, Santoro A. Inflammaging: a new immune-metabolic viewpoint for age-related diseases. Nat Rev Endocrinol. 2018;14(10):576–90. 30046148 10.1038/s41574-018-0059-4

[18] Rea IM, Gibson DS, McGilligan V, McNerlan SE, Alexander HD, Ross OA. Age and Age-Related diseases: role of inflammation triggers and cytokines. Front Immunol. 2018;9:586. 29686666 10.3389/fimmu.2018.00586 PMC5900450

[19] Tacke F, Randolph GJ. Migratory fate and differentiation of blood monocyte subsets. Immunobiology. 2006;211(6–8):609–18. 16920499 10.1016/j.imbio.2006.05.025

[20] Puchta A, Naidoo A, Verschoor CP, Loukov D, Thevaranjan N, Mandur TS, et al. TNF drives monocyte dysfunction with age and results in impaired anti-pneumococcal immunity. PLoS Pathog. 2016;12(1):e1005368. 26766566 10.1371/journal.ppat.1005368 PMC4713203

[21] Sadeghi HM, Schnelle JF, Thoma JK, Nishanian P, Fahey JL. Phenotypic and functional characteristics of circulating monocytes of elderly persons. Exp Gerontol. 1999;34(8):959–70. 10673149 10.1016/s0531-5565(99)00065-0

[22] Kratofil RM, Kubes P, Deniset JF. Monocyte Conversion during inflammation and Injury. Arterioscler Thromb Vasc Biol. 2017;37(1):35–42. 27765768 10.1161/ATVBAHA.116.308198

[23] Kimball A, Schaller M, Joshi A, Davis FM, denDekker A, Boniakowski A, et al. Ly6C(hi) blood Monocyte/Macrophage drive chronic inflammation and impair Wound Healing in Diabetes Mellitus. Arterioscler Thromb Vasc Biol. 2018;38(5):1102–14. 29496661 10.1161/ATVBAHA.118.310703 PMC5920725

[24] Shaw TN, Houston SA, Wemyss K, Bridgeman HM, Barbera TA, Zangerle-Murray T, et al. Tissue-resident macrophages in the intestine are long lived and defined by Tim-4 and CD4 expression. J Exp Med. 2018;215(6):1507–18. 29789388 10.1084/jem.20180019 PMC5987925

[25] Sampathkumar NK, Bravo JI, Chen Y, Danthi PS, Donahue EK, Lai RW, et al. Widespread sex dimorphism in aging and age-related diseases. Hum Genet. 2020;139(3):333–56. 31677133 10.1007/s00439-019-02082-w PMC7031050

[26] WHO. Global Health Estimates. Life expectancy and leading causes of death and disability 2022 https://www.who.int/data/gho/data/themes/mortality-and-global-health-estimates

[27] Lee S, Kim M, Lee Y, Kim J, Jang HC, Cho B, et al. The effect of sex and physical frailty on incident disability after 2 years among community-dwelling older adults: KFACS study. BMC Geriatr. 2022;22(1):588. 35840889 10.1186/s12877-022-03263-5 PMC9288076

[28] Hagg S, Jylhava J. Sex differences in biological aging with a focus on human studies. Elife. 2021;10. 10.7554/eLife.63425 PMC8118651 33982659

[29] Kennedy AE, Cook L, Breznik JA, Cowbrough B, Wallace JG, Huynh A et al. Lasting changes to circulating leukocytes in people with mild SARS-CoV-2 infections. Viruses. 2021;13(11). 10.3390/v13112239 PMC8622816 34835045

[30] WHO. Healthy ageing and functional ability 2022 https://www.who.int/news-room/questions-and-answers/item/healthy-ageing-and-functional-ability

[31] Loukov D, Karampatos S, Maly MR, Bowdish DME. Monocyte activation is elevated in women with knee-osteoarthritis and associated with inflammation, BMI and pain. Osteoarthritis Cartilage. 2018;26(2):255–63. 29128509 10.1016/j.joca.2017.10.018

[32] Verschoor CP, Kohli V, Balion C. A comprehensive assessment of immunophenotyping performed in cryopreserved peripheral whole blood. Cytometry B Clin Cytom. 2018;94(5):662–70. 28378896 10.1002/cyto.b.21526

[33] Breznik JA, Jury J, Verdu EF, Sloboda DM, Bowdish DME. Diet-induced obesity alters intestinal monocyte-derived and tissue-resident macrophages and increases intestinal permeability in female mice independent of tumor necrosis factor. Am J Physiol Gastrointest Liver Physiol. 2023;324(4):G305–21. 36749921 10.1152/ajpgi.00231.2022

[34] Lozoya-Agullo I, Araujo F, Gonzalez-Alvarez I, Merino-Sanjuan M, Gonzalez-Alvarez M, Bermejo M, et al. Usefulness of Caco-2/HT29-MTX and Caco-2/HT29-MTX/Raji B coculture models to predict intestinal and colonic permeability compared to Caco-2 monoculture. Mol Pharm. 2017;14(4):1264–70. 28263609 10.1021/acs.molpharmaceut.6b01165

[35] Team RC. R: A language and environment for statistical computing. Vienna, Austria2017 https://www.R-project.org/

[36] Bohnke JR. Explanation in causal inference: methods for mediation and interaction. Q J Exp Psychol (Hove). 2016;69(6):1243–4. 28190383 10.1080/17470218.2015.1115884

[37] Tingley D, Yamamoto T, Hirose K, Keele L, Imai K. mediation: R Package for Causal Mediation Analysis. R package version 4.4.2 2013 http://CRAN.R-project.org/package=mediation

[38] Op het Veld LP, van Rossum E, Kempen GI, de Vet HC, Hajema K, Beurskens AJ. Fried phenotype of frailty: cross-sectional comparison of three frailty stages on various health domains. BMC Geriatr. 2015;15:77. 26155837 10.1186/s12877-015-0078-0 PMC4496916

[39] Care CTFoPH. Asymptomatic Thyroid Dysfunction - Clinican Summary 2019 [ https://canadiantaskforce.ca/asymptomatic-thyroid-dysfunction-clinician-summary/#:~:text=Thyroid%20dysfunction%20(i.e.%2C%20hypothyroidism%20or,years%20of%20age%20(16%25).

[40] Albert PR. Why is depression more prevalent in women? J Psychiatry Neurosci. 2015;40(4):219–21. 26107348 10.1503/jpn.150205 PMC4478054

[41] Untersmayr E, Brandt A, Koidl L, Bergheim I. The intestinal barrier dysfunction as driving factor of inflammaging. Nutrients. 2022;14(5). 10.3390/nu14050949 PMC8912763 35267924

[42] Fasano A, Not T, Wang W, Uzzau S, Berti I, Tommasini A, et al. Zonulin, a newly discovered modulator of intestinal permeability, and its expression in coeliac disease. Lancet. 2000;355(9214):1518–9. 10801176 10.1016/S0140-6736(00)02169-3

[43] Sapone A, de Magistris L, Pietzak M, Clemente MG, Tripathi A, Cucca F, et al. Zonulin upregulation is associated with increased gut permeability in subjects with type 1 diabetes and their relatives. Diabetes. 2006;55(5):1443–9. 16644703 10.2337/db05-1593

[44] Lau E, Marques C, Pestana D, Santoalha M, Carvalho D, Freitas P, et al. The role of I-FABP as a biomarker of intestinal barrier dysfunction driven by gut microbiota changes in obesity. Nutr Metab (Lond). 2016;13:31. 27134637 10.1186/s12986-016-0089-7 PMC4851788

[45] Sorgdrager FJH, Naude PJW, Kema IP, Nollen EA, Deyn PP. Tryptophan Metabolism in Inflammaging: from biomarker to therapeutic target. Front Immunol. 2019;10:2565. 31736978 10.3389/fimmu.2019.02565 PMC6833926

[46] Cervenka I, Agudelo LZ, Ruas JL, Kynurenines. Tryptophan’s metabolites in exercise, inflammation, and mental health. Science. 2017;357(6349). 10.1126/science.aaf9794 28751584

[47] Dehhaghi M, Kazemi Shariat Panahi H, Heng B, Guillemin GJ. The gut microbiota, Kynurenine Pathway, and Immune System Interaction in the development of Brain Cancer. Front Cell Dev Biol. 2020;8:562812. 33330446 10.3389/fcell.2020.562812 PMC7710763

[48] Ho J, Chan H, Liang Y, Liu X, Zhang L, Li Q, et al. Cathelicidin preserves intestinal barrier function in polymicrobial sepsis. Crit Care. 2020;24(1):47. 32041659 10.1186/s13054-020-2754-5 PMC7011568

[49] Chow JY, Li ZJ, Wu WK, Cho CH. Cathelicidin a potential therapeutic peptide for gastrointestinal inflammation and cancer. World J Gastroenterol. 2013;19(18):2731–5. 23687409 10.3748/wjg.v19.i18.2731 PMC3653146

[50] Fang X, Nong K, Wang Z, Jin Y, Gao F, Zeng Q, et al. Human cathelicidin LL-37 exerts amelioration effects against EHEC O157:H7 infection regarding inflammation, enteric dysbacteriosis, and impairment of gut barrier function. Peptides. 2022;159:170903. 36370932 10.1016/j.peptides.2022.170903

[51] Zhang L, Yu J, Wong CC, Ling TK, Li ZJ, Chan KM, et al. Cathelicidin protects against Helicobacter pylori colonization and the associated gastritis in mice. Gene Ther. 2013;20(7):751–60. 23254369 10.1038/gt.2012.92

[52] Ren Z, Pan LL, Huang Y, Chen H, Liu Y, Liu H, et al. Gut microbiota-CRAMP axis shapes intestinal barrier function and immune responses in dietary gluten-induced enteropathy. EMBO Mol Med. 2021;13(8):e14059. 34125490 10.15252/emmm.202114059 PMC8350901

[53] Wong CC, Zhang L, Wu WK, Shen J, Chan RL, Lu L, et al. Cathelicidin-encoding Lactococcus lactis promotes mucosal repair in murine experimental colitis. J Gastroenterol Hepatol. 2017;32(3):609–19. 27470075 10.1111/jgh.13499

[54] Ghosh S, Lertwattanarak R, Garduno Jde J, Galeana JJ, Li J, Zamarripa F, et al. Elevated muscle TLR4 expression and metabolic endotoxemia in human aging. J Gerontol Biol Sci Med Sci. 2015;70(2):232–46. 10.1093/gerona/glu067 PMC4311182 24846769

[55] Li Y, Lee PY, Sobel ES, Narain S, Satoh M, Segal MS, et al. Increased expression of FcgammaRI/CD64 on circulating monocytes parallels ongoing inflammation and nephritis in lupus. Arthritis Res Ther. 2009;11(1):R6. 19144150 10.1186/ar2590 PMC2688236

[56] Marsh SA, Arthur HM, Spyridopoulos I. The secret life of nonclassical monocytes. Cytometry A. 2017;91(11):1055–8. 29077270 10.1002/cyto.a.23280

[57] De Schepper S, Verheijden S, Aguilera-Lizarraga J, Viola MF, Boesmans W, Stakenborg N, et al. Self-maintaining gut macrophages are essential for intestinal homeostasis. Cell. 2018;175(2):400–15. e13. 30173915 10.1016/j.cell.2018.07.048

[58] Droessler L, Cornelius V, Markov AG, Amasheh S. Tumor necrosis factor Alpha effects on the Porcine Intestinal Epithelial Barrier Include enhanced expression of TNF receptor 1. Int J Mol Sci. 2021;22(16). 10.3390/ijms22168746 PMC8395858 34445450

[59] Seidler S, Zimmermann HW, Bartneck M, Trautwein C, Tacke F. Age-dependent alterations of monocyte subsets and monocyte-related chemokine pathways in healthy adults. BMC Immunol. 2010;11:30. 20565954 10.1186/1471-2172-11-30 PMC2910032

[60] Ghosh SS, Wang J, Yannie PJ, Ghosh S. Intestinal barrier dysfunction, LPS translocation, and Disease Development. J Endocr Soc. 2020;4(2):bvz039. 32099951 10.1210/jendso/bvz039 PMC7033038

