# Supplementary material for: Monocyte-driven inflamm-aging reduces intestinal barrier function in females
Source: Immun Ageing. 2024 Sep 30;21:65. doi: 10.1186/s12979-024-00469-6 (PMC11440997; doi:10.1186/s12979-024-00469-6)
Supplement: Supplementary file 1 — Supplementary Material 1 [file 12979_2024_469_MOESM1_ESM.docx]

| **Additional File S1:** Fluorophore-conjugated monoclonal antibodies used for flow cytometry | | | | |
| --- | --- | --- | --- | --- |
| **Cell Surface Marker** | **Fluorophore** | **Clone** | **Company** | **Cat No.** |
| **Human myeloid staining** |  |  |  |  |
| CD45 | BV510 | HI30 | BioLegend | 304036 |
| CD16 | PE-Cy7 | CB16 | eBioscience | 25-0168-42 |
| CD14 | BV421 | M5E2 | BioLegend | 301830 |
| CCR2 | PE | K036C2 | BioLegend | 357205 |
| CD11b | APC | ICRF44 | BD Biosciences | 561015 |
| HLA-DR | PerCPCy5.5 | LN3 | eBioscience | 45-9956-42 |
| CX3CR1 | FITC | 2A9-1 | Cedarlane | D070-4 |
| CD15 | BV650 | SSEA-1 | BioLegend | 323033 |
| CD3 | AF700 | UCHT1 | BD Biosciences | 557943 |
| CD56 | AF700 | 5.1H11 | BioLegend | 362522 |
| CD19 | AF700 | HIB19 | eBioscience | 56-0199-42 |
|  |  |  |  |  |
| **Mouse myeloid staining- peripheral blood** |  |  |  |  |
| CD45 | eF450 | 30-F11 | invitrogen | 48-0451-82 |
| CD11b | PECy7 | M1/70 | invitrogen | 25-0112-82 |
| Ly6C | AF488 | HK1.4 | BioLegend | 128022 |
| CCR2 | PE | 475301 | R&D systems | FAB5538P |
| F4/80 | APC | BM8 | eBioscience | 17-4801-82 |
| CD3 | AF700 | 17A2 | invitrogen | 56-0032-82 |
| CD19 | AF700 | eBio1D3 | invitrogen | 56-0193-82 |
| NK | AF700 | PK136 | invitrogen | 56-5941-82 |
| Ly6G | AF700 | 1A8 | BioLegend | 127622 |
| CX3CR1 | BV650 | SA011F11 | BioLegend | 149033 |
