# Supplementary figures and images for: Monocyte-driven inflamm-aging reduces intestinal barrier function in females
Source: Immun Ageing. 2024 Sep 30;21:65. doi: 10.1186/s12979-024-00469-6 (PMC11440997; doi:10.1186/s12979-024-00469-6)

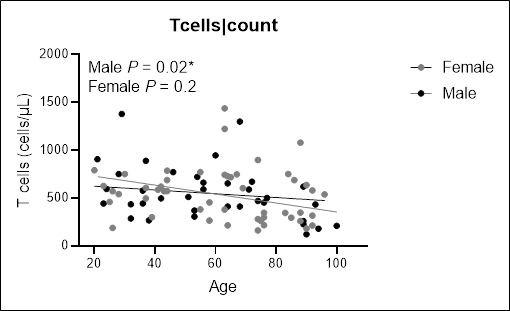


Additional File S2. T cell numbers decrease in the peripheral blood of males with age.

Supplement: Supplementary file 2 — Supplementary Material 2 [file 12979_2024_469_MOESM2_ESM.docx]

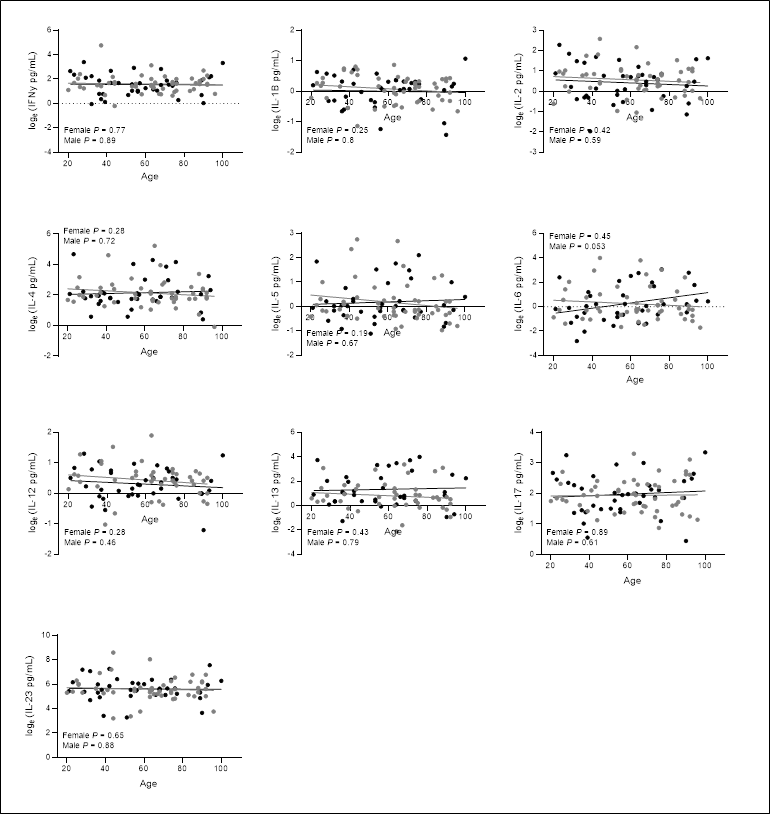


Additional File S3. Peripheral blood cytokines across the life-course in healthy agers.

Supplement: Supplementary file 3 — Supplementary Material 3 [file 12979_2024_469_MOESM3_ESM.docx]

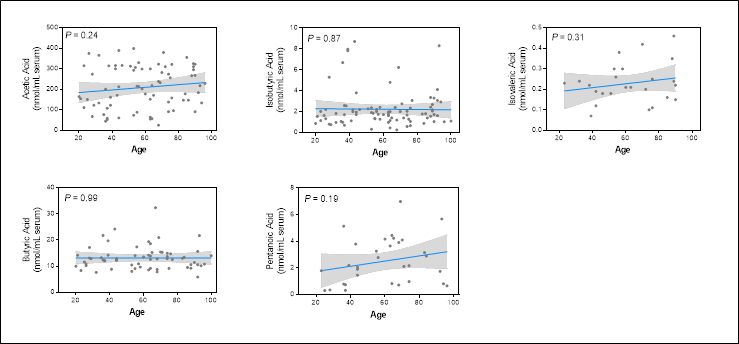


Additional File S4. Short-chain fatty acids in the peripheral blood of healthy agers.

Supplement: Supplementary file 4 — Supplementary Material 4 [file 12979_2024_469_MOESM4_ESM.docx]
